# Assessment of #TheDress With Traditional Color Vision Tests: Perception Differences Are Associated With *Blueness*

**DOI:** 10.1177/2041669518764192

**Published:** 2018-03-27

**Authors:** Claudia Feitosa-Santana, Margaret Lutze, Pablo A. Barrionuevo, Dingcai Cao

**Affiliations:** Albert Einstein Israelite Hospital, São Paulo, SP, Brazil; Federal University of ABC, São Bernardo do Campo, SP, Brazil; Department of Biological Sciences, DePaul University, Chicago, IL, USA; Institute of Research in Light, Environment and Vision, National University of Tucumán—National Scientific and Technical Research Council, San Miguel de Tucumán, Argentina; Department of Ophthalmology and Visual Sciences, University of Illinois at Chicago, IL, USA

**Keywords:** #TheDress, color, development, individual differences, light, lightness/brightness, perception

## Abstract

Based on known color vision theories, there is no complete explanation for the perceptual dichotomy of #TheDress in which most people see either white-and-gold (WG) or blue-and-black (BK). We determined whether some standard color vision tests (i.e., color naming, color matching, anomaloscope settings, unique white settings, and color preferences), as well as chronotypes, could provide information on the color perceptions of #TheDress. Fifty-two young observers were tested. Fifteen of the observers (29%) reported the colors as BK, 21 (40%) as WG, and 16 (31%) reported a different combination of colors. Observers who perceived WG required significantly more blue in their unique white settings than those who perceived BK. The BK, blue-and-gold, and WG observer groups had significantly different color preferences for the light cyan chip. Moreland equation anomaloscope matching showed a significant difference between WG and BK observers. In addition, #TheDress color perception categories, color preference outcomes, and unique white settings had a common association. For both the bright and dark regions of #TheDress, the color matching chromaticities formed a continuum, approximately following the daylight chromaticity locus. Color matching to the bright region of #TheDress showed two nearly distinct clusters (WG vs. BK) along the daylight chromaticity locus and there was a clear cutoff for reporting WG versus BK. All results showing a significant difference involved blue percepts, possibly due to interpretations of the illuminant interactions with the dress material. This suggests that variations in attributing *blueness* to the #TheDress image may be significant variables determining color perception of #TheDress.

## Introduction

#TheDress phenomenon that began after Caitlin McNeil posted a picture of the dress on February 26, 2015 has provoked much discussion within the color vision science community and in the general public because it is unclear why people perceive the dress image, for the most part, in one of two very different ways. When observing the original image of the dress, most people report either seeing a white-and-gold (WG) dress or a blue-and-black (BK) dress. Most color vision scientists agree that the lighting conditions in the original image, the unique properties of the dress materials, and potential camera white balancing, somehow combine in a special way that produces this unique perceptual phenomenon (Brainard & Hurlbert, 2015; Gegenfurtner, Bloj, & Toscani, 2015; Hesslinger & Carbon, 2016; Lafer-Sousa, Hermann, & Conway, 2015; Toscani & Gegenfurtner, 2017; Vemuri, Bisla, Mulpuru, Varadharajan, & Varadarajan, 2016; Winkler, Spillmann, Werner, & Webster, 2015; Witzel, Racey, & Regan, 2017).

A fundamental question about #TheDress phenomenon concerns the underlying mechanisms that determine which perceptual category an observer belongs to; for example, those perceiving a WG dress or BK dress. Possible explanations that have been proposed include individual differences in blue–yellow perceptual asymmetry (Winkler et al., 2015), lightness/brightness over chromaticity computations (Gegenfurtner et al., 2015), color constancy (Brainard & Hurlbert, 2015; Gegenfurtner et al., 2015), internalized preferences for cool or warm illuminants that are associated with chronotypes (Lafer-Sousa et al., 2015; Wallisch, 2017), pupil sizes (Vemuri et al., 2016), differences in higher level cognition (Schlaffke et al., 2015), and the density of the macular luteal pigment (Rabin, Houser, Talbert, & Patel, 2016). More recently, other explanations have included inferred position of the light source (Chetverikov & Ivanchei, 2016), implicit assumptions about the illuminant (Witzel et al., 2017), differences in sensitivity to contextual cues (Toscani & Gegenfurtner, 2017), one-shot learning influences (Daoudi, Kunchulia, & Herzog, 2017), sensitivity to the low spatial frequency content (Dixon & Shapiro, 2017), and genetic and environmental factors (Mahroo, Williams, Hossain, Yonova-doing, & Hammond, 2017). However, none of these potential explanations provide a complete account of the underlying mechanisms and some explanations lack empirical evidence.

Overall, perceptual differences could be attributed to variations in low-level (e.g., retinal) mechanisms to higher level (cortical) mechanisms. For example, it is well known that color vision can be affected by anomalies at the receptoral level, such as in the common X-linked color vision variations, and the perception of #TheDress could be due to variations at the receptoral level (macular pigment optical density; Rabin et al., 2016). On the other hand, many color vision properties, such as adaptation phenomena or color preferences, have been ascribed to higher order neural processing and therefore higher order factors could also account for the dress phenomenon (Brainard & Hurlbert, 2015; Gegenfurtner et al., 2015; Schlaffke et al., 2015; Toscani & Gegenfurtner, 2017; Winkler et al., 2015).

One approach for understanding #TheDress color phenomenon is to analyze interindividual color perceptions of the dress in relation to color vision variations at different processing stages measured with well-known psychophysical methods. We, therefore, designed a study using some tests that have been commonly applied to investigate individual differences in color perception. We employed color naming of #TheDress, color matching to the image of #TheDress, anomaloscope color matching, unique white settings, and surface color preference testing. Some of these tests are known for displaying large individual differences at the receptoral level (Alpern & Moeller, 1977; Carroll, Neitz, & Neitz, 2002; Dartnall, Bowmaker, & Mollon, 1983; Winderickx et al., 1992), unique white settings (Bosten, Beer, & MacLeod, 2015; Webster & Leonard, 2008; Werner & Schefrin, 1993), and color preferences (Ellis & Ficek, 2001; Miyahara, Szewczyk, McCartin, & Caldwell, 2004; Palmer & Schloss, 2010; Taylor & Franklin, 2012) and were therefore considered to possibly be more informative. Of the five color tests, two of them (color naming and color matching) were used to characterize the perception of #TheDress. The other three tests were independent of #TheDress perception and were chosen because they can reflect visual processing at different stages: The anomaloscope matching provides information on the earliest stage of color processing, at the receptoral level (Cao, 2017); the unique white point is considered the equilibrium point of postreceptoral mechanisms, possibly set at the lateral geniculate nucleus (Bosten et al., 2015); the color preference test is meant to provide information on higher level processing in that color preference has been tied to cultural preferences as an evolutionary advantage (Palmer & Schloss, 2010).

The goal of this study was to clarify whether individual differences in these measurements could help to understand the dissimilarities between the groups of observers with different color perceptions of #TheDress; for example, WG and BK. We assessed whether #TheDress color perception is associated with unique white settings that fall on the blue–yellow axis since the novel perceptual phenomenon of #TheDress has been related to daylight; that is, the settings localized very closely to the daylight locus in relative Troland space and the cerulean line (Lafer-Sousa et al., 2015; Pearce, Crichton, Mackiewicz, Finlayson, & Hurlbert, 2014; Winkler et al., 2015). In addition, we included a morningness–eveningness questionnaire to check the chronotype hypothesis (Lafer-Sousa et al., 2015).

## Methods

### Observers

Fifty-two young observers (26 females and 26 males; age 22.38 [*M*] ± 3.20 [*SD*]), who were students at the School of the Art Institute of Chicago or friends of students, participated in the study. All had normal or corrected-to-normal acuity and normal color vision as assessed with the Ishihara plates and an anomaloscope. All observers were aware of the Internet “viral” phenomenon of #TheDress but were naïve as to our research purposes. Each observer gave written informed consent. This research protocol was in accordance with the Declaration of Helsinki and was approved by the Institutional Review Board at The School of the Art Institute of Chicago.

### Apparatus and Calibration

Stimuli used for color naming, color matching, unique white settings, and color preferences were generated on an iMac computer using the software Psychopy (Peirce, 2007) and were presented on a SONY Trinitron 21-inch calibrated CRT color monitor (1280 by 1024 pixels, 75 Hz). Linearization and spectrum calibration of the monitor was completed with a PR-670 Spectroradiometer (PhotoResearch Inc., Chatsworth, CA). The anomaloscope matching task was carried out using the Oculus HMC Anomaloscope (OCULUS Optikgeräte GmbH, Wetzlar, Germany).

### Overall Procedure

Every observer performed all of the psychophysical tests during one session in a dark room in the following sequence: anomaloscope matching, color preference testing, unique white setting, dress color naming, and dress color matching. Anomaloscope testing was performed first to screen for observers with color vision deficiencies. Then, color preferences and unique white settings were tested as second and third to avoid any potential impact of seeing #TheDress colors in relation to color preferences and unique white settings. Finally, observers completed color naming and color matching of #TheDress. The chronotype questionnaire was completed by email to shorten the test session. The protocol lasted approximately 30 min to 50 min, and only the anomaloscope test was monocular. The details of the tests are described in the following sections.

### Color Vision Tests

#### Anomaloscope matching

Anomaloscope testing included Rayleigh matching for assessing relative L- and M-cone spectral sensitivity functions and Moreland matching for assessing S-cone spectral sensitivity functions. In both matching procedures, a circular field (2°) was presented that was horizontally divided into two halves: an upper half and a bottom half. In the Rayleigh matching, a spectral “yellow” (589 nm) comparison light was presented in the bottom field, and a mixture of “green” (549 nm) and “red” (666 nm) spectral primaries was presented in the upper field. Observers were asked to adjust the red–green ratio and brightness of the yellow to make the two semicircular fields appear identical (Cao, 2017). In the Moreland matching procedure, a bicolor test field (“cyan” 480 nm and “yellow” 589 nm) was presented in the bottom field and matched to a mixture of two primaries (“blue” 436 nm and “green” 490 nm) in the upper field. Observers adjusted the blue–green ratio and brightness of the test field to make the two fields appear identical. Following the instrument manual, both tests were performed twice for each eye. All of the results for the observers included in the study were in the normal range for each match, confirming normal color vision. The between-trial and between-eye variations were relatively small, as between-trial and between-eye variation accounted for 8.5% of the total variance for the Rayleigh color matching data, 1.3% of the total variance for the Rayleigh brightness matching, 6.4% of the total variance for the Moreland color matching, and 0.2% of the total variance for the Moreland brightness matching. To simplify the analysis, the results from the two eyes for each observer were averaged for further analysis.

#### Surface color preference testing

**Figure 1.**
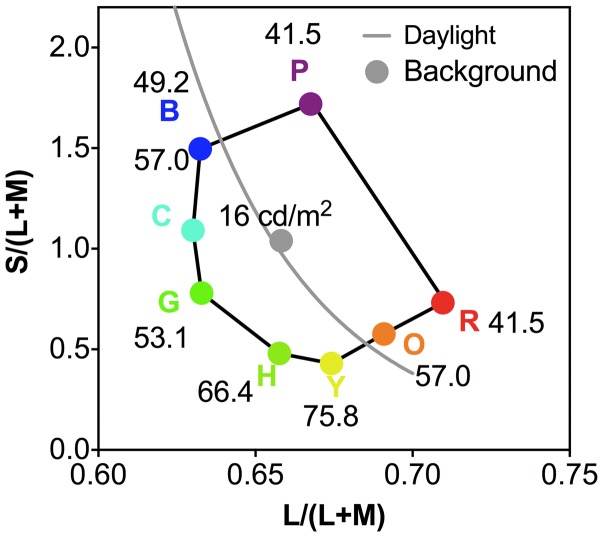
The eight light colored stimuli (Palmer & Schloss, 2010) used in the color preference test, including red (R), orange (O), yellow (Y), chartreuse (H), green (G), cyan (C), blue (B), and purple (P) with the luminance in cd/m^2^ indicated for each color chip.

For the color preference test, we used eight light colored stimuli (Palmer & Schloss, 2010), including red (R), orange (O), yellow (Y), chartreuse (H), green (G), cyan (C), blue (B), and purple (P) (Figure 1). According to Palmer and Schloss (2010), the four cuts (saturated, muted, light, and dark) differed in their saturation and lightness levels; light cuts were those that were approximately halfway between each saturated color and the Munsell value of 9 and chroma of 1 for the same hue. We chose light colors as a previous study (Gegenfurtner et al., 2015) reported that the bright region of the #TheDress image largely determined #TheDress color.

We used the same CIE 1931 x,y chromaticities as in the Palmer and Schloss (2010) study for the test colors—see Figure 1 for the corresponding L/(L+M) and S/(L+M) chromaticities in the relative cone Troland space (Smith & Pokorny, 1996), which was derived from the MacLeod and Boynton (1979) chromaticity space, and the S/(L+M) chromaticity was normalized for an equal energy light as 1.0 (Smith & Pokorny, 1996). The background was spatially uniform and had the (x,y) chromaticity of (0.312, 0.318), which was the same chromaticity as in the study by Palmer and Schloss. Due to the monitor gamut, we used a background luminance of 16 cd/m^2^ instead of 19.26 cd/m^2^, which was used in their original study. We scaled the luminance of each color stimulus by a factor of 0.83 (=16/19.26) to maintain the same luminance contrast as in the original study (Weber contrasts 159%–315%).

The preference for each color was rated three times and, therefore, there were 24 trials in total for each observer. The order of the color presentations was random. At each trial, one color was presented in a 10°× 10° square field in the monitor center and observers rated how much they liked the color on a scale from −5 (*not at all*) to + 5 (*very much*) by sliding the mouse cursor along a response scale and clicking to record their responses. The ratings from the three repetitions were averaged and then were normalized by a linear scaling such that, for each observer, the most preferred color was set as 100 and the least preferred color was −100.

#### Unique white settings

For the unique white settings, a 1° circular field with a dark background was presented in the center of the monitor. The luminance was set at 16 cd/m^2^, the same background luminance as color preference testing. The chromaticity was specified in relative cone Troland space (Smith & Pokorny, 1996; see the Surface Color Preference Testing section for more details). At the beginning of each trial, the stimulus was given a random chromaticity near equal energy white [L/(L+M)= 0.667, S/(L+M) = 1.00]. During the experiment, the observer adjusted the stimulus along L/(L+M) and S/(L+M) axes using the upward, downward, left, and right buttons on a USB Logitech Precision gamepad, sensed by the computer, to achieve a white perception (i.e., nonreddish, nongreenish, nonbluish, and nonyellowish). Pressing the upward or downward buttons increased or decreased the S/(L+M) chromaticity, respectively. Pressing the right or left buttons increased or decreased the L/(L+M) chromaticity, respectively. A separate button was designated to confirm the unique settings. Each observer repeated the unique white setting three times and the results were averaged for further analysis. The observers were told that each button (up, down, right, and left) had a color change associated with more blue (up button), yellow (down button), red (right button), or green (left button) and that it was necessary to move the buttons (up, down, right, and left) in order to get the whitest white possible; once the whitest white was achieved, the observers would have to press another button (central) to confirm.

#### #TheDress color naming

For color naming of dress perceptions, #TheDress original image (6.8° × 10.3°) was presented on the center of the monitor. The observers were verbally asked “What are the colors of the dress?” The observers were free to respond with any color names. Luminance and chromatic distributions of #TheDress image were provided elsewhere (Gegenfurtner et al., 2015; Lafer-Sousa et al., 2015). The link to #TheDress image that was used here is: https://upload.wikimedia.org/wikipedia/en/a/a8/The_Dress_%28viral_phenomenon%29.png

#### Color matching to #TheDress

For the color matching test, the same image of #TheDress was presented again on the center of the monitor. To avoid bias, we did not identify the “bright” or “dark” regions of the dress and, therefore, the observers chose their own regions as bright or dark for color matching. The response colors provided by an observer in the color naming test were used in the color matching test. For example, if they indicated BK in color naming of #TheDress, the instructions for color matching were first to identify either blue or black as the bright region of the dress. Invariably, for BK observers, blue was the bright region and black was the dark region, while for WG observers, the white was the bright region and gold was the dark region. The observers were then asked to match the color of the bright region of the dress image by pressing buttons on the gamepad to adjust for color (L/(L+M) and S/(L+M)) and luminance (L+M) of a circular matching field (diameter 3°), which was set on the left side of the dress image for the bright region. In the sequence, the observers were also asked to match the color and luminance of the darker region of the dress using the same procedure, which was set on the right side. The distance between the border of the color matching field and the dress image was approximately two inches (5.7°). No instructions were given to the observers about eye movements. The starting chromaticity and luminance of the matching field were randomly chosen. The color and luminance were then adjusted to match #TheDress as close as possible. This color matching was repeated three times for both the bright and dark regions and was then averaged for further analysis. There was no time limit for this task.

#### Chronotype Questionnaire

After the testing sessions were completed, the morningness–eveningness (chronotype) self-assessment questionnaire (MEQ-SA) (Horne & Ostberg, 1976) was mailed to all the participants. Observers were asked to answer the 19 questions on the questionnaire. Each question was rated between 0 (*evening-preference*) and 6 (*morning-preference*). The total score was computed to indicate a morningness chronotype (59–69, moderate and 70–86, definite morning) or an eveningness chronotype (31–41, moderate and 16–30, definite evening). In total, 46 of 52 participants completed the chronotype survey.

### Statistical Analyses

Chi-squared tests were used to compare categorical variables (sex and ethnicity) among the observer groups that reported different dress image colors. One-way analyses of variance (ANOVAs) were used to compare continuous variables (age, chronotype score, anomaloscope tests, unique white setting, or color matching) among the observer groups and, if significant, Duncan’s post hoc test was then used for pairwise comparisons. A two-way repeated measures ANOVA was used to assess the effect of the observer group and color chip on color preferences followed by Duncan’s post hoc test. Canonical discriminant analysis was used to determine whether color-matching data in the bright or dark regions of the dress image determined the dress color names. Finally, Pearson’s correlational analyses were used to assess the association between anomaloscope tests, unique white settings, color preferences, and dress-color matching results.

## Results

### #TheDress Color Naming

**Figure 2.**
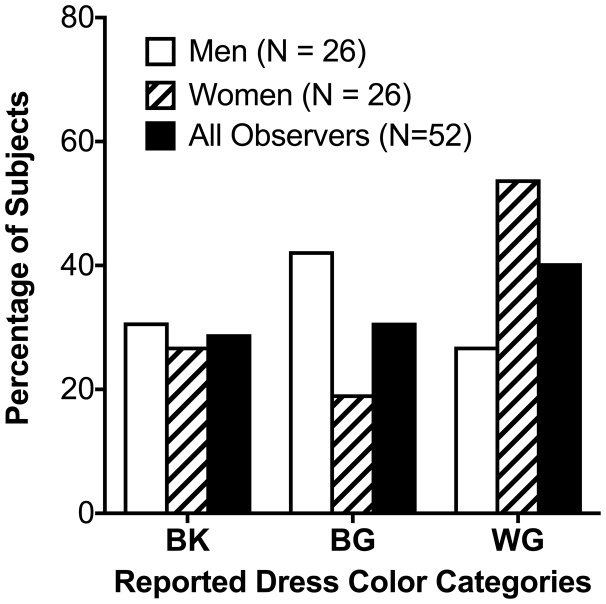
Distribution of #TheDress color perceptions: The percentages of observers who named the dress image as blue-and-black (BK), blue-and-gold (BG), or white-and-gold (WG), for men (*N* = 26), women (*N* = 26), and all observers together (*N* = 52).

For our sample (*N* = 52), 15 (29%) observers reported the colors of the dress image as BK and 21 (40%) reported the colors as WG. The remaining 16 observers (31%) reported the dress image color as a combination of blue and gold, specifically as “blue and gold,” “blue and brown,” “grayish-blue and grayish-yellow,” “white and goldish-brown,” “blue and orange,” “blue and greenish-brown,” “blue and yellowish-gray,” “light blue and gold,” “blue golden and golden brown,” goldish-green and whitish-blue,” “blue and brownish-olive,” “white and goldish-yellow,” and “periwinkle gold.” We treated these observers as one category, namely, blue-and-gold (BG) (Figure 2). The reported dress image color was not significantly associated with age, *F*(2, 49) = 0.57, *p* = .57, ethnicity, χ^2^(2) = 4.18, *p* = .38, or chronotype score, *F*(2, 49) = 0.11, *p* = .898. However, the reported dress image color was marginally associated with sex, χ^2^(1) = 4.65, *N* = 52, *p* = .098, with a trend for more women to report WG than men (Figure 2).

### Color Matching to #TheDress

**Figure 3.**
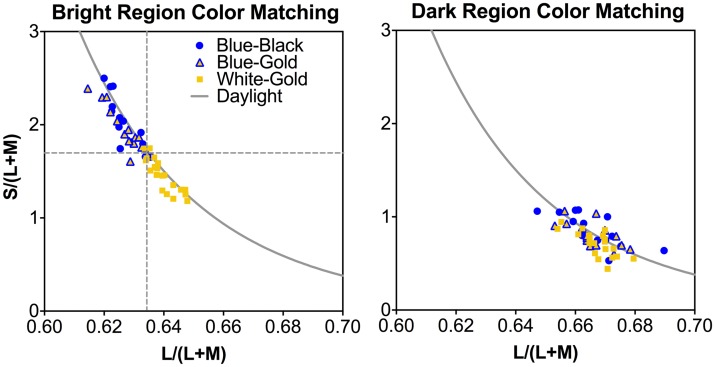
Individual color matching to #TheDress results: Individual matching L/(L+M) and S/(L+M) results for the bright (left panel) and dark (right panel) regions of the dress image. The dashed gray line in the left panel largely represent the cutoff L/(L+M) and S/(L+M) for reporting white-and-gold versus blue-and-black and blue-and-gold. In each panel, the gray line represents the daylight locus.

**Figure 4.**
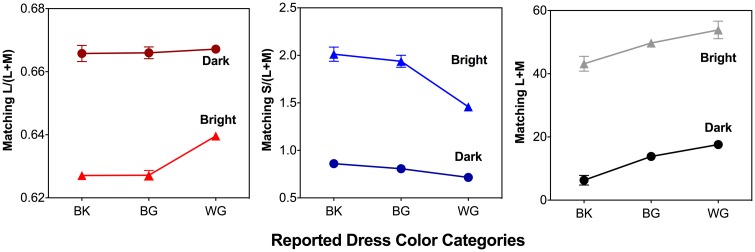
Group mean color matching to #TheDress results: Mean L/(L+M) (left panel), S/(L+M) (middle panel) and (L+M) (right panel) matching results for the bright (triangles) and dark (circles) regions of the dress image. The unit for luminance (L+M) is cd/m^2^. There is no unit for L/(L+M) or S/(L+M). Error bars are ±SEM. For the bright region, WG observer group had higher L/(L+M) and lower S/(L+M) compared to BG or BK observer groups. For the dark region, the three observer groups did not differ in L/(L+M), while the WG observer group had lower S/(L+M) compared to BK observer group. For both bright and dark regions of the dress, the matching luminance (L+M) was significantly different among the BK, BG, and WG groups. BK: blue-and-black; BG: blue-and-gold; WG: white-and-gold.

The L/(L+M), S/(L+M) and (L+M) matching results for the bright (perceived as white or blue) and dark (perceived as gold or black) regions of the dress image for the BK, BG, and WG observer groups are shown in Figures 3 and 4. For both the bright and dark regions, the matching chromaticities formed a continuum, approximately following the daylight chromaticity locus (Figure 3; left panel bright region, right panel dark region). For the bright region of the dress image, two nearly distinct clusters were formed and there was a clear cutoff chromaticity for reporting WG versus BK or BG (Figure 3, left panel, dashed line), as matching L/(L+M) was higher for WG observer group compared to BG or BK observer groups, *F*(2,49) = 33.7, *p* < .001, while the WG observer group reported lower S/(L+M) than the BG or BK observer group, *F*(2, 49) = 30.17, *p* < .001 (Figure 4). For the dark region, there was no clear cutoff chromaticity for the WG, BK, or BG groups (Figure 3, right panel), as matching L/(L+M) did not differ among the three observer groups, *F*(2, 49) = 0.14, *p* = .89, while the WG observer group reported lower S/(L+M), *F*(2, 49) = 4.26, *p* = .02, than the BK observer group (Figure 4). For both bright and dark regions of the dress, the matching luminance (L+M) was significantly different among the BK, BG and WG groups, bright region: *F*(2, 49) = 5.07, *p* = .01; dark region: *F*(2, 49) = 31.39, *p* < .001 (Figure 4, right panel). Post hoc tests indicated that, in the bright region, the WG observers had higher matching luminance (*M* = 53.86, *SD* = 12.76) than the BK group (*M* = 43.17, *SD* = 9.14), while in the dark region the WG observers had higher matching luminance (*M* = 17.57, *SD* = 2.95) than the BG group (*M* = 14.07, *SD* = 3.72), which, in turn, had higher matching luminance than the BK group (*M* = 6.32, *SD* = 5.96). These matching results indicated that both the perceived color and brightness in the bright and dark regions were important in determining color naming of #TheDress. Both color and lightness in the bright region could differentiate the WG group from the BK and BG groups, while only the lightness in the dark region could differentiate the three color groups WG, BG, and BK. Canonical discriminant analysis indicated that using the matching of L/(L+M), S/(L+M), and (L+M) in both regions could correctly classify 87% of the reported #TheDress color names among the observers, with the bright region L/(L+M) and S/(L+M) matching and the dark region luminance (L+M) match playing the most important role in determining #TheDress color names, as evidenced by large discriminant loadings (≥ 0.43) for these measurements in the bright region. BK: blue-and-black; BG: blue-and-gold; WG: white-and-gold.

### Anomaloscope Matching

**Figure 5.**
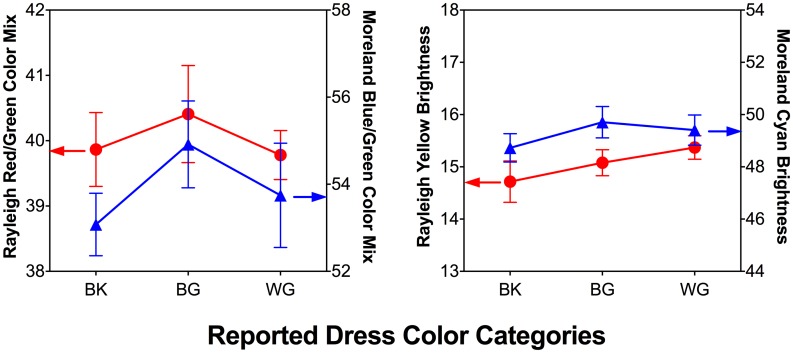
The anomaloscope color matching results (Rayleigh and Moreland, left panel) and associated brightness settings results (right panel). Red circles indicate mean red–green match (Rayleigh) settings for each group and blue triangles indicate mean blue–yellow match (Moreland) settings for each group. The x-axis represents BK, BG, and WG observer groups. Error bars are ± SEM. None of the anomaloscope matching results differed among the WG, BK, and BG observer groups; Moreland color mixture ratios were significantly correlated with matches of the dark region of the dress. BK: blue-and-black; BG: blue-and-gold; WG: white-and-gold.

Rayleigh and Moreland color mixture ratios (Figure 5, left panel) or associated brightness settings in anomaloscope matching (Figure 5, right panel) did not differ among the WG, BK, and BG observer groups, *F*(2, 49) ≤ 1.35, *p* ≥ .27. When the association between the anomaloscope measurements and dress color matching was considered, the Moreland color mixture ratio was significantly correlated with matches of the dark region of the dress S/(L+M), *r*(51) = −.32, *p* = .02.

### Unique White Settings

**Figure 6.**
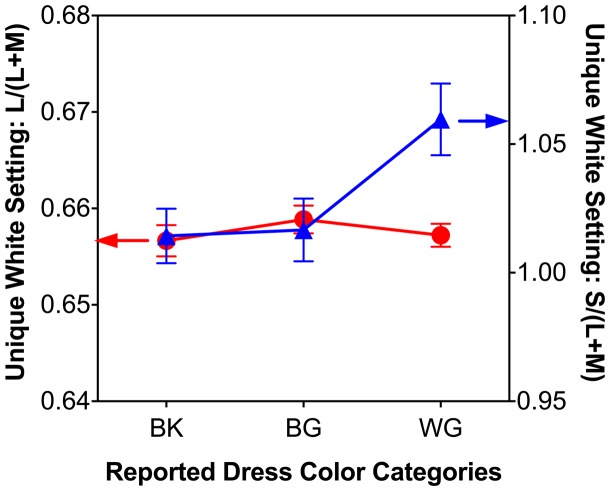
The unique white settings results for the BK, BG, and WG observer groups. Red circles represent L/(L+M) (left axis) and blue triangles represent S/(L+M) (right axis) for each group. Error bars are ± SEM. The WG observer group had significantly higher S/(L+M) settings (more blue) in their unique white settings. BK: blue-and-black; BG: blue-and-gold; WG: white-and-gold.

In the unique white settings, the WG group had significantly higher unique white S/(L+M) settings, meaning more blue was added than in the BG or BK groups (Figure 6; right axis), *F*(2, 49) = 4.38, *p* = .02. The L/(L+M) results (Figure 6; left axis), however, did not differ among the three groups, *F*(2, 49) = 0.50, *p* = .61. Also, the unique white S/(L+M) settings were significantly correlated with matching L/(L+M), *r*(50) = .30, *p* = .03, and S/(L + M), *r*(50) = −.329, *p* = .04, in the bright region of the dress, with higher unique white S/(L+M) settings associated with higher matching L/(L+M) and lower matching S/(L+M) values in the bright region (Figure 3, left panel). BK: blue-and-black; BG: blue-and-gold; WG: white-and-gold.

### Surface Color Preference Testing

**Figure 7.**
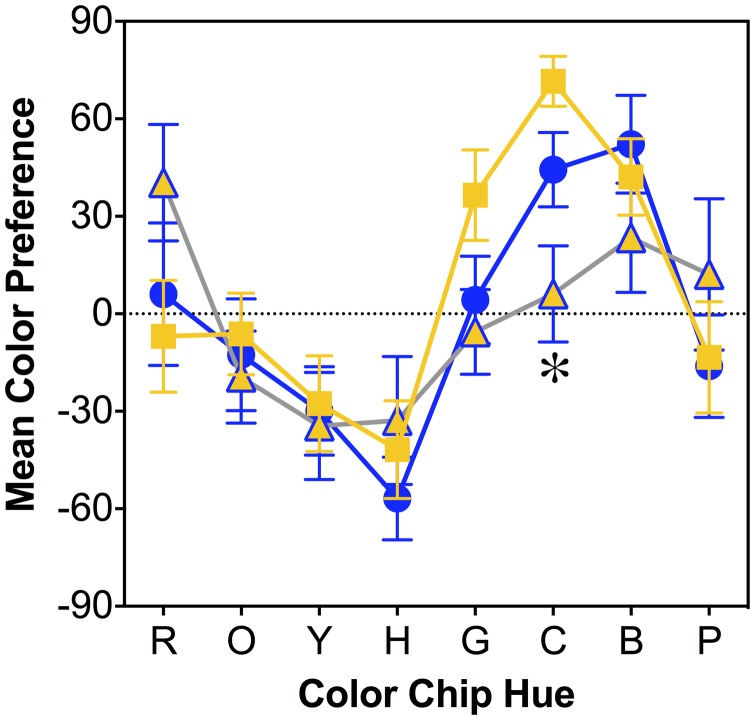
Surface color preferences results for the light color chips within the BK, BG, and WG observer groups. The yellow square symbols refer to the WG group, blue circles refer to the BK group and the yellow triangles with a blue outline refer to the BG group. Error bars are ±SEM. *Significance at Duncan’s adjusted significance level (i.e., *p* < .01 = 0.05/8) for comparing color preferences among observer groups for a single color chip. Color preference for the light cyan was significantly different among WG, BK, and BG observer groups.

Overall, the color preferences for the light colors followed a similar pattern as in the study by Palmer and Schloss (2010). The BK, BG, and WG observer groups had significantly different color preferences for the light cyan chip (Figure 7); *F*(2, 49) = 9.00, *p* < .001; Duncan’s post hoc test: WG > BK > BG. Correlational analysis indicated that the light green and light cyan preferences were significantly correlated with dress image color matching data in the bright region of the dress with higher preferences for light green and light cyan associated with higher matching L/(L+M), light green versus L/L(L+M): *r* = .481, *p* < .001; light cyan versus L/(L+M): *r* = .444, *p* = .001, and lower matching S/(L+M), light green versus S/L(L+M): *r* = −.466, *p* < .001; light cyan versus S/(L+M): *r* = −.375, *p* = .006, in the bright region of the dress image. In addition, the unique white S/(L+M) settings were significantly correlated with the preference ratings of light green (*r* = .325, *p* = .019) and light cyan (*r* = .396, *p* = .004). In our sample, color preferences for the eight color stimuli were not associated with sex, *F*(1, 50) ≤ 1.16, *p* ≥ .29.

## Discussion

We evaluated the perception of #TheDress in association with some commonly used color vision tests, including anomaloscope matching, unique white settings, color naming and matching to #TheDress, and surface color preferences. Our study found significant associations with the perception of #TheDress for unique white settings, color matching with #TheDress bright and dark regions, Moreland equation color matching, and surface color preferences. We also tested the chronotype hypothesis (Lafer-Sousa et al., 2015) and the #TheDress reported colors were not associated with the chronotype scores. All results in our study that showed a significant difference among the groups involved blue percepts, suggesting that variations in attributing “blueness” to the #TheDress image, possibly due to interpretations of the illuminant interactions with the dress material, may be the significant variable determining the perception of #TheDress.

We found the distribution of color naming as follows: 40% reported WG, 29% reported BK, and the remaining 31% reported different color naming that fit the BG category. One study (Lafer-Sousa et al., 2015) reported a different distribution of color naming of #TheDress: approximately 40% reported WG, 50% reported BK, and 10% reported BG. However, the sum of the BK and BG percentages in this study (Lafer-Sousa et al., 2015;60%) was the same as the sum of the BK and BG percentages in our study (60%). Most of the observers in both studies were not naïve regarding the image; however, most of the observers in our study were art students and were very interested in the discussion about #TheDress and color perception. Experience became a predictor in their logistic regression (Lafer-Sousa et al., 2015), suggesting that experience in terms of exposure to #TheDress image put the observer into the nonnaïve category and shaped the language used to describe the dress and possibly the perception of it. Therefore, learning may influence the perception of #TheDress although this suggestion is not aligned with the one-shot learning hypothesis (Daoudi et al., 2017).

Other studies also assessed the frequency distribution of #TheDress colors. One study (Witzel et al., 2017) found a more similar prevalence of BG observers compared to our study, which followed this distribution: 34% reported WG, 25% reported BK, and 40% reported a combination of naming that fit the BG category. One study (Vemuri et al., 2016) found a very similar distribution between WG and BK: 51.5% reported WG and 48.5% reported BK. One study (Mahroo et al., 2017) with 446 twins (85% female) found approximately 64% perceived #TheDress as WG, 27% as BK, and 9% as a combination of other colors. In this sample, 70% were not naïve, while the remaining 30% were naïve and had never seen the image previously. These striking differences in the frequency of #TheDress reported colors described in different studies in different regions of the world may indicate that cues (amount of sunlight, length of daylight, and time spent indoors or outdoors) influence the perception of the #TheDress (Chetverikov & Ivanchei, 2016).

Two studies (Lafer-Sousa et al., 2015; Mahroo et al., 2017) reported that dress image colors were associated with age. Therefore, some aging factors, such as lens yellowing, could be an important consideration. In our study, our observers were young (18–34 years old) and age-related lens yellowing was not a likely source of variation in the perception of the dress since significant lens changes only occur after the age of 60 years (Pokorny, Smith, & Lutze, 1987). In addition, it is necessary to consider that the white neutral locus does not vary with age, possibly due to normalization mechanisms (Delahunt, Webster, Ma, & Werner, 2004), and if the aging process influences the color perception of #TheDress there must be another explanation and not the lens yellowing effect.

Our color matching to #TheDress results showed that the perceived colors in the bright and dark regions of #TheDress image largely followed the natural daylight locus in relative cone Troland space (Figure 3), in agreement with previous studies (Brainard & Hurlbert, 2015; Gegenfurtner et al., 2015; Lafer-Sousa et al., 2015; Toscani & Gegenfurtner, 2017; Winkler et al., 2015; Witzel et al., 2017). In our study, WG observers had higher matching luminances than the BK and BG groups, suggesting that perceived brightness played an important role in dress image color naming. This result was consistent with a study (Gegenfurtner et al., 2015) that had a much smaller sample (*N* = 15) in which interobserver variation in the perception of the dress image was shown to be due to differences in lightness. Another study (Toscani & Gegenfurtner, 2017) also found a difference in lightness but not in chromaticity. However, different from these studies, we found a clear cutoff in color matching for the bright region of the dress with lower S/(L+M) and higher L/(L+M) settings for the WG group compared to the BK and BG group, suggesting that the dichotomy of #TheDress is not exclusively explained by the perceived brightness but also by chromaticity, which is in agreement with another study (Lafer-Sousa et al., 2015). The settings for the WG group formed a distinct cluster that was different from the cluster along the natural daylight locus for the BK and BG groups. For the dark region of the dress, the WG group had lower S/(L+M) settings compared to the BK and BG groups (Figure 4). This result showed that, in our study, the matching chromaticity also played an important role in the color perception of #TheDress. Our discriminant analysis identified that bright region matching chromaticities and dark region matching luminances were the most informative in the dress color categorization.

Our results showed that observers who perceived #TheDress as WG had higher S/(L+M) in the unique white settings compared to the other groups (BK and BG). This means that more blue was added by the WG observers to determine the unique white setting in comparison to the BK and BG observers. Therefore, if unique white settings reveal the appearance of white in general, #TheDress should appear white for those with more blue in their unique white settings. Unique white settings are thought to represent internal normalization of cone opponency mechanisms, and it is well documented that great individual differences have been shown to exist in the white neutral locus settings along the locus of variations in daylight illumination, likely due to adaption to environmental lighting conditions (Bosten et al., 2015; Mollon, 2006; Webster & Leonard, 2008). In agreement with these results, we found that the Moreland color mixture ratio was significantly correlated with matches to the dark region of the dress S/(L+M), suggesting the perceived dress color is at least partially related to the S-cone function. One study (Winkler et al., 2015) suggested that WG observers were more likely to have larger blue–yellow asymmetries, which may be related to individual differences in the internal normalization of cone opponency mechanisms (Walraven & Werner, 1991), and our study provides evidence that it is indeed a result of this internal normalization since we found differences in the white neutral locus as well as the Moreland equation ratio that are associated with the dichotomy of #TheDress color perception.

One group (Winkler et al., 2015) studied the inversion of #TheDress color content. Observers were not adapted directly to the dress image itself, but instead to alternating blue and yellow fields. Their results showed no asymmetry in the strength of afterimages elicited by blue or yellow biased adapting stimuli, suggesting that the blue–yellow perceptual asymmetry might be linked to a higher order process rather than processing at a receptoral level. These results are in agreement with our unique white settings since higher unique white settings were associated with the perception of #TheDress, meaning that more blue was added to determine the unique white in association with perceiving the dress image as WG. Also, aligned with our results and this study (Winkler et al., 2015), another study (Toscani & Gegenfurtner, 2017) that made use of a probe in the front and in the background of #TheDress, applied a similar method, although different from our unique white settings. Observers were asked to adjust the probe to look white and the WG observers tended to produce bluer adjustments to the probe compared to the BK observers, suggesting that WG perceivers are more sensitive to contextual cues compared to BK perceivers.

Some studies (Bosten et al., 2015; Pearce et al., 2014; Werner & Schefrin, 1993) showed that color discrimination is poorest in the blue–yellow direction that falls along the daylight locus. It is possible that interobserver variations in daylight locus blue–yellow discrimination exist that may contribute to the dichotomy in #TheDress color perceptions. It was therefore hypothesized that internalized preferences for cool or warm illuminants that are associated with chronotypes may explain the dress color phenomenon, such that older people and women were more likely to have a daytime chronotype and were more likely to perceive the dress as WG than men and younger observers (Lafer-Sousa et al., 2015). However, the reported dress image colors in our study were not associated with the chronotype scores, in agreement with another study (Aston & Hurlbert, 2017). This means that the chronotypes of our observers could not explain the differences in color perceptions of #TheDress.

Our study showed that color perception of #TheDress was associated with some surface color preferences. Observers in all groups had significantly different preferences for light cyan. Also, higher preferences for light green and light cyan were associated with higher L/(L+M) color matching values and lower S/(L+M) color matching values to the bright region of #TheDress. Color preferences are, in general, highly influenced by familiarity and exposure effects (Jacoby, Kelley, Brown, & Jasechko, 1989; Whittlesea, Jacoby, & Girard, 1990). A study (Winkler et al., 2015) suggested that familiarity may not play a role in the perception of the #TheDress. However, it is possible that, in reverse, we also may perceive what we prefer, which could be related to the cultural influences on color preference (Palmer & Schloss, 2010). This is aligned with other studies (Wallisch, 2017; Witzel et al., 2017) in which they found evidence that the perceived colors of the dress image depend on an assumption about the illumination, implying the power of unconscious inference. Our correlational analysis showed that the preferences for light green and light cyan were associated with unique white S/(L+M) settings for color perceptions of the bright region of the dress, which in turn were associated with the reported color of #TheDress. This means that color preference outcomes were associated with the unique white settings as well as the color perception of #TheDress, and observers with higher preferences for light green and light cyan were in the cluster of observers with more blue in their unique white settings. Surface color preferences in our sample did not reveal sex differences, in agreement with the original study (Palmer & Schloss, 2010), although some studies have shown sex differences in color preferences (Hurlbert & Ling, 2007; Sorokowski, Sorokowska, & Witzel, 2014; Taylor & Franklin, 2012; Yokosawa, Schloss, Asano, & Palmer, 2016). Therefore, the limits and extent that sex differences influence color preferences remain unknown. Regarding color perception of #TheDress, our study suggested that sex differences in color preferences do not play an important role.

In our study, we analyzed color preferences as surface color preferences, while another study (Lafer-Sousa et al., 2015) queried their observers about illuminant colors being appropriate in the dress image and proposed an illuminant preference hypothesis. The blueness of the bright region of the dress may vary among observers depending on how much blue they attribute to the illuminant versus the reflectance from the surface of the dress. Another study (Toscani & Gegenfurtner, 2017) tested the same thing with a probe, checking how much blue was added to the illuminant in the front and the background of the image, and arrived at the same conclusion as our study. Also, these studies were not aligned with another study (Witzel et al., 2017) that presented results suggesting that WG observers do not simply discount bluish colors more than BK observers, implying that WG observers do not have a blue bias in general.

We suggest consideration of a developmental mechanism based on experiences with sunlight (warm illuminant) and skylight (cool illuminant, in opposition to Lafer-Sousa et al., 2015) during infancy/adulthood as a possible basis for the dichotomy in colloquially reported color perceptions of #TheDress. The color vision system continues to develop after birth (Cornish, Hendrickson, & Provis, 2004), especially during the first year of life (Brown, 1990). It has been suggested that there is a developmental process that occurs from early infancy to adulthood that sets the individual’s color preferences (Adams, 2013; Palmer & Schloss, 2010) and interindividual differences in exposure to UV-B during infancy may result in interindividual differences in color preferences or unique hues along the daylight blue/yellow axis in color (Miyahara et al., 2004). It has also been suggested that natural scene experiences and light exposure during early infancy and childhood may shift the balance of the blue–yellow opponency mechanism to affect unique white settings (Bosten et al., 2015; Cornish et al., 2004). Studies mentioned in the Introduction section (Brainard & Hurlbert, 2015; Gegenfurtner et al., 2015; Lafer-Sousa et al., 2015; Toscani & Gegenfurtner, 2017; Winkler et al., 2015) and our color matching results all hinted at perceptions of #TheDress having a relation to sunlight/skylight perceptions. This novel phenomenon discovered by chance over the Internet may suggest that there are differences in how the color vision system develops based on whether a person grows up and/or has long-term experience in an environment in which they are regularly exposed to sunlight or in an environment with more limited exposure to the outdoors such that they have few experiences with sunlight that underlie the perceptions of #TheDress. This developmental hypothesis is speculative and requires further study.
